# Stage of disease in hepatitis B virus infection in Zambian adults is associated with large cell change but not well defined using classic biomarkers

**DOI:** 10.1093/trstmh/trx077

**Published:** 2018-01-25

**Authors:** Bright Nsokolo, Anne Kanunga, Edford Sinkala, Kanekwa Zyambo, Dia Kumwenda, David Chama, Gabriel Muyinda, Michael Vinikoor, Samreen Ijaz, Richard Tedder, Ali Khalifa A Elmdaah, Meleri Jones, Clarence Chiluba, Victor Mudenda, Robert D Goldin, Graham Foster, Paul Kelly

**Affiliations:** 1 TROPGAN, Department of Internal Medicine, University of Zambia School of Medicine, Lusaka, Zambia; 2 Zambia National Blood Transfusion Service, University Teaching Hospital, Lusaka, Zambia; 3 University of Alabama at Birmingham, Birmingham, Alabama, USA; 4 Blood-Borne Virus Unit, Microbiology Services, Public Health England, Colindale, UK; 5 Barts and The London School of Medicine and Dentistry, Queen Mary University of London, London, UK; 6 Department of Pathology and Microbiology, University Teaching Hospital, Lusaka, Zambia; 7 Department of Hepatology, Imperial College London, London, UK

**Keywords:** Africa, Hepatitis B, Hepatocellular carcinoma, liver biopsy

## Abstract

**Background:**

Hepatocellular malignancy in young adults is a prominent feature of hepatitis B virus (HBV) infection in southern Africa. Here we report a cross-sectional study of liver pathology correlated with biomarkers in adults with HBV infection in Zambia.

**Methods:**

We analysed liver biopsies from Zambian patients with persistent HBV infection.

**Results:**

We analysed 104 patients with HBV infection and evidence of liver disease. We obtained liver biopsies from 53 adults; of these, 12 (23%) were hepatitis B e antigen seropositive. The genotype was evenly distributed between A and E. One biopsy showed malignancy. Stage was 3 or more in 11 of 52 (21%) biopsies free of malignancy and lobular inflammation was found in 50 (94%). Neither alanine aminotransferase (ALT) nor the γ-glutamyl transferase:platelet ratio (GPR) were correlated with the stage of disease but were correlated with total Ishak score (ρ=0.47, p=0.0004 and ρ=0.33, p=0.02, respectively). Large cell change was observed in 10 of 11 biopsies with fibrosis stage 3 or more and 16 of 41 with early disease (p=0.005). Serum α-fetoprotein was elevated, although still within the normal range, in patients with large cell change (median 3.6 [interquartile range {IQR} 1.6–5.1]) compared with those without (1.7 [IQR 1.0–2.8]; p=0.03). Neither ALT nor GPR predicted large cell change.

**Conclusions:**

Large cell change was common in young HBV-infected adults in Zambia. Only serum α-fetoprotein was identified as a biomarker of this phenotype.

## Introduction

Persistent infection with the hepatitis B virus (HBV) is endemic in Africa, involving many millions of infected people. Early studies from South Africa^1^ showed a high incidence of hepatocellular carcinoma (HCC) in African men, with a further increased incidence in rural communities. A number of factors may predispose to the high burden of malignancy in Africans with persistent HBV infection, including environmental factors such as aflatoxin B1, viral factors (genotype A1 is associated with a higher incidence of malignancy) and presumably, host genetic influences.^2,3^ However, we still do not have a full understanding of the mechanisms underlying the high incidence of HCC in African men and, of importance, known interventions that may reduce the cancer rates are not consistently and properly deployed.

Effective antiviral therapy for persistent HBV infection is becoming widely available throughout the world. Treatment with potent antiviral drugs (such as tenofovir and entecavir) allows long-term suppression of viral replication with clear evidence of reversal of liver fibrosis.^4^ The impact of prolonged antiviral therapy on HCC remains to be determined, although many believe that any impact will be modest. Persistent HBV infection follows a prolonged course with an initial period of high viral replication and minimal liver inflammation (the so-called immunotolerant phase) followed by episodes of liver inflammation that often lead to viral suppression and the development of an ‘inactive’, hepatitis B e antigen (HBeAg)-negative phase of infection. This ‘inactive carrier’ phase may persist for decades, but in many it is followed by viral reactivation and the development of HBeAg-negative disease. Given the fluctuating natural history of persistent HBV infection and the prolonged periods of inactivity, experts agree that antiviral therapy should only be introduced during periods of active disease, when the greatest benefits are likely to accrue.^5–7^ The European Association for the Study of the Liver (EASL) recommends that patients be considered for treatment when they have HBV DNA levels >2000 IU/mL, have serum alanine aminotransferase (ALT) levels above the upper limit of normal and evidence of moderate to severe necroinflammation and/or at least moderate fibrosis on liver biopsy. However, these guidelines were developed in Asia and developed countries and their value in Africa has been questioned by recent studies from The Gambia suggesting that conventional diagnostic approaches may be suboptimal.^8,9^ There is also uncertainty about optimal scoring systems for liver biopsies, and several are available. Here we focus on the Ishak score, a well-known score focused on inflammation.

Zambia has a high prevalence of persistent HBV infection, with estimates ranging from 4 to 8%; data from the Zambian National Blood Transfusion Service suggest that prevalence varies across the country. Zambia is bordered by countries known to have a high incidence of hepatocellular carcinoma,^2^ but the true incidence in Zambia is unknown. Clinical experience confirms that, in common with adjacent high-prevalence countries, cancer occurs in young adults.

In southern Africa, it is common to diagnose hepatocellular carcinoma in men with chronic HBV in their third decade of life. Identifying markers of malignant susceptibility in such patients is of great importance. Large cell change may be such a marker, but its significance is unclear. The increased cell size and nucleolar abnormalities suggest that these changes have malignant potential and could be considered as dysplastic. In this study we looked at the prevalence of these changes.

We recently initiated a trial of antiviral therapy in Zambia and here we report that in patients with persistent HBV who were assessed as potential participants for the study, we found a high prevalence of histological damage, chiefly large cell change, associated with biochemical parameters that would not normally lead to consideration of therapy. These data indicate that persistent HBV in Africa may lead to potentially pre-malignant change in the absence of classical features indicating a need for antiviral therapy. These findings suggest that a reassessment of treatment criteria in Africa will be required to reduce the burden of this disease.

## Study setting and methods

This study was carried out in the University Teaching Hospital (UTH), Lusaka, Zambia, a secondary and tertiary referral hospital in the capital city. Recruitment was conducted from January 2014 to September 2015. Patients were identified from two sources: blood donors whose donations were identified as seropositive for hepatitis B surface antigen and patients attending the clinics or on the wards of UTH who were identified with any evidence of liver dysfunction or inflammation. The data reported here are drawn from the baseline data collected from patients recruited to the StepHep study (www.isrctn.com, ISRCTN40785133), which was designed to test the hypothesis that monotherapy with tenofovir could safely be followed by monotherapy with lamivudine if full viral suppression had been achieved during tenofovir treatment; the results of this longitudinal study will be published elsewhere. Approval for this study was granted by the University of Zambia Biomedical Research Ethics Committee on 24 May 2013 (005-02-13) and by the Zambia Medicines Regulatory Authority on 14 August 2013 (CT040/13) and a favourable ethical opinion was also granted by the National Health Service National Research Ethics Service Committee (London City and East) on 10 October 2013. Written evidence of consent was obtained from all participants.

### Patient recruitment

Patients were offered inclusion in the study if they were hepatitis B surface antigen (HBsAg) seropositive at a single time point. If they agreed to inclusion, they were further assessed clinically, with blood drawn for ALT and viral load, and were assessed for liver biopsy. The inclusion criteria included ALT >35 IU/L, HBV DNA >2000 IU/mL and evidence of necroinflammation on liver biopsy. When a transient elastography instrument was made available in June 2015, this was also performed and 5 kPa was added as an inclusion criterion. Exclusion criteria included HIV infection, a history of alcohol abuse, a history of anti-tuberculous drug ingestion or virological evidence of active HCV or HEV infection or serological evidence of previous infection with schistosomiasis.

### Study procedures

Once identified as having HBV infection and any evidence of liver inflammation, patients were approached and interviewed by a study physician (BN) or nurse (AK) and informed consent was sought. The patients were then interviewed, blood was collected and they were then prepared for liver biopsy, with measurement of the international normalized ratio and platelet count included in all cases. When available, transient elastography was performed using a Fibroscan instrument (Echosens, Paris, France). Two values of cut-off were used: 7.2 kPa, as recommended by the manufacturer for chronic HBV infection, and a more exploratory cut-off of 5 kPa, which would be expected to exclude fibrosis with greater stringency.

### Virological assessment

Plasma was obtained from venous blood samples by centrifugation and aliquots were stored at −80°C so that no more than one freeze–thaw cycle was required during analysis. HBeAg and antibodies to HDV were detected by enzyme-linked immunosorbent assay (ELISA; Diasorin, Dartford, UK). For viral load quantification, DNA was extracted from plasma using Blood DNA minikits (Qiagen, Hilden, Germany) and amplified by real-time polymerase chain reaction using standard primers (forward: GTGTCTGCGGCGTTTTATCA; reverse GACAAACGGGCAACATACCTT) in a PRISM 7500 Sequence Detection System (Applied Biosystems, Foster City, CA, USA), with HBV standards, murine cytomegalovirus and known HBV-positive controls in every run. For genotyping, HBV DNA was extracted from 33 plasma samples using a QIAamp MinElute Virus Spin Kit (Qiagen). A 940-bp amplicon, using previously described primers^10^ (HBV_1F 5′TAGGACCCCTGCTCGTGTTACAGG and HBV_4R 5′GAAAGGCCTTGTAAGTTGGCG), was amplified using Phusion High-Fidelity DNA polymerase. Sanger sequencing using the above primers was performed by Source Bioscience (Cambridge, UK). Sequences were aligned using BioEdit against HBV genotype A and E reference sequences (GenBank accession codes X02763.1 and X75657.1, respectively) and submitted to the HBVdb website (http://hbvdb.ibcp.fr) to confirm genotyping. Surface antigen was quantified using the Abbott Architect system (Abbott Diagnostics, Lake Forest. IL. USA). Schistosomiasis antibodies were tested by ELISA (Scimedx,Dover, NJ, USA).

### Liver biopsies

Biopsies were obtained under local anaesthesia using a 16G Menghini biopsy needle and immediately placed in formalin-saline prior to paraffin embedding and preparation of haematoxylin and eosin–stained sections. Biopsies were evaluated by two expert pathologists (VM and RDG) and both stage and Ishak score were evaluated. In each case, at least four portal tracts were evaluated and most cores were at least 4 cm long. In order to help interpret the significance of large cell change when detected, 30 samples of liver tissue taken post-mortem from adults killed in road traffic accidents were reviewed (RDG). Also reviewed were 100 consecutive biopsies from HBV-infected patients at St Mary’s Hospital (London, UK).

### Data analysis

Data are reported as discrete or continuous (non-Gaussian) variables (median and interquartile range [IQR]) and non-parametric statistics were used throughout. Hypothesis testing employed Fisher’s exact test for categorical variables and Kruskal–Wallis test for continuous variables, and correlation was estimated using Spearman’s rank correlation coefficient (ρ). All authors had access to the study data and reviewed and approved the final manuscript.

## Results

Of 347 adults who were identified to be seropositive for HBsAg, 104 HIV-seronegative patients were identified as having evidence of liver inflammation, either by ALT >35 IU/L or by a transient elastography reading >5 kPa; 243 patients had neither of these characteristics or were HIV co-infected (for a flow diagram, see the Supplementary material). From the remaining 104 patients, 69 underwent needle liver biopsy, but results are available for only 68, as one was lost in processing. Reasons for not performing biopsy are shown in Supplementary Table 1. Baseline characteristics are shown in relation to HBeAg status (Table 1) or eligibility for treatment (Supplementary Table 2). Two patients were confirmed as having HCC, one with high α-fetoprotein (455–500 kU/L) and one on histology in a patient who was not initially suspected of having cancer. Two patients were confirmed as having cirrhosis on histology. Transient elastography data were available for 64 patients (Supplementary Table 3).

**Table 1.** Baseline characteristics, by ALT and e antigen status

	e Antigen status		
	Positive (n=25)	Negative (n=79)	p-Value
Sex (male:female)	20:5	68:11	0.60
Age (y)	23 (19–30)	31 (27–42)	0.0002
BMI (kg/m^2^)	21.2 (20.1–23.0)	22.9 (20.2–25.7)	0.22
Alcohol use currently, n	3	20	0.43
Schistosomiasis, n	1	3	—
HDV antigen positive, n	0	1	—
ALT	72 (45–95)	36 (25–50)	0.001
Platelet count (×10^9^/L)	189 (148–257)	224 (196–272)	0.07
Viral load (log units/ml)	7.9 (5.3–8.3)	2.9 (2.4–3.8)	0.0001
γ-glutamyl transferase	40 (23–83)	42 (26–71)	0.97
HBsAg concentration	12 745 (8994–18 337)	6018 (2538–12 982)	0.003
Fibroscan (kPa)^a^	7.1 (6.0–18.9) [n=12]	6.2 (5.7–7.1) [n=52]	0.054
Genotype	type A: 7 (44%)	type A: 10 (59%)	0.49
	type E: 9 (56%)	type E: 7 (41%)	

Data presented as median (IQR) unless stated otherwise. The p-values shown were estimated by Fisher’s exact test for proportions or Kruskal–Wallis test for continuous variables.

^a^The smaller number of people with Fibroscan results in the high ALT group is a consequence of starting the study without Fibroscan; when it became available we changed the recruitment criteria to include all ALT values. Normal range of ALT <35 IU/L; normal range of γ-glutamyl transferase <51 IU/L.

### Liver biopsy findings and biomarkers of disease severity

**Figure 1.**
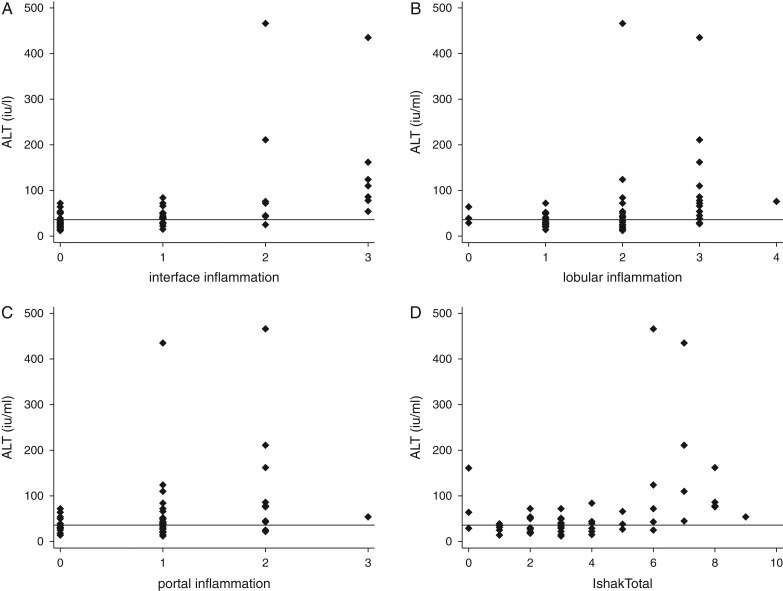
Relationships between serum ALT and Ishak scores on histology. Three components of the Ishak score are shown, together with the total score: (A) interface inflammation (Spearman’s ρ=0.64, p<0.001); (B) lobular inflammation (ρ=0.41, p=0.002); (C) portal inflammation (ρ=0.33, p=0.01); (D) total Ishak score (ρ=0.47, p=0.004).

Of note, 11 of 52 patients had evidence of fibrosis on histology (stage 3 or greater). Stage was not related to age (ρ=0.003, p=0.99), HBe antigenaemia (Table 2) or body mass index (ρ=−0.01, p=0.95). Lobular inflammation was prominent, particularly in those who were e antigen positive (Table 2). Interface and portal inflammation were generally mild and confluent necrosis was uncommon (Table 2). Of the 11 patients with fibrosis on biopsy, 10 had serum ALT >35 IU/L (p=0.03) and 10 had a viral load of ≥2000 IU/L (p=0.007). ALT was correlated with the total Ishak score (ρ=0.47, p=0.004; Figure 1) and more particularly with interface inflammation (ρ=0.64, p<0.0001) and lobular inflammation (ρ=0.42, p=0.002). However, the predictive value was weak (Figure 1), such that ALT was elevated in six patients with Ishak scores ≤2 and was not elevated in five patients with Ishak scores ≥4. The initial viral load was correlated with Ishak score (ρ=0.36, p=0.009). To assess histological evidence of need for treatment, we used standard criteria: Ishak score ≥4 or stage 3 or greater. Using standard criteria for eligibility for treatment^6^ (ALT >35 IU/L and viral load >2000 IU/L), 38 patients would be correctly classified; however, 9 patients with histological evidence of a need for treatment would have been missed and 6 patients unnecessarily treated. The γ-glutamyl transferase:platelet ratio (GPR) was elevated in 9 of 11 biopsies with significant fibrosis (stage 3 or greater) and 21 of 35 without (p=0.28), but it was significantly associated with eligibility for treatment (Supplementary Table 2). Transient elastography readings were not correlated with either histological stage (p=0.78 by non-parametric test for trend) or Ishak score (ρ=−0.03, p=0.87).

**Table 2.** Histology by eAg status

		eAg seronegative	eAg seropositive	p-Value
Stage	0	8	2	0.32
	1	12	2	
	2	13	3	
	3	4	2	
	4	2	2	
	6	0	1	
Interface inflammation	0	17	4	0.03
	1	13	2	
	2	7	1	
	3	2	5	
Confluent necrosis	0	38	12	1.00
	1	1	0	
Lobular inflammation	0	2	0	0.004
	1	17	0	
	2	13	5	
	3	6	7	
	4	1	0	
Portal inflammation	0	9	2	0.41
	1	21	7	
	2	9	2	
	3	0	1	
Ishak score total, median (IQR) [range]		5.5 (3.5–7.5) [2–9]	3.0 (2.0–5.0) [0–8]	0.02

The p-values shown were estimated by Fisher’s exact test for proportions or Kruskal–Wallis test for continuous variables.

**Table 3.** Large cell change in liver biopsies

	Large cell change present (n=26)	Large cell change absent (n=27)	p-Value
Sex (male:female)	20:6	25:2	0.14
Age (y)	30 (23–40)	28 (23–41)	0.77
ALT (IU/L)	53 (29–86)	38 (27–52)	0.16
e Antigen, n (%)	9 (35)	3/26 (12)	0.10
HBV viral load (log IU/L)	3.8 (2.9–6.1)	3.2 (2.4–4.1)	0.11
α-fetoprotein (kU/ml)	3.6 (1.6–5.1)	1.7 (1.0–2.8)	0.03
Fibroscan (kPa)	6.3 (5.3–7.4)	6.5 (6.0–7.6)	0.66
Stage, n			
0	2	9	0.02
1	7	7	
2	7	9	
3	5	1	
4	4	0	
5	0	0	
6	0	0	
Ishak score total	4 (3–6)	3 (2–4)	0.01
Eligible for treatment, n (%)	13 (50)	9 (33)	0.27
HBsAg concentration (IU/ml)	7833 (2784–13 994) [n=23]	5345 (59–13 804) [n=15]	0.28

Data are presented as median (IQR) unless otherwise noted. The p-values shown were estimated by Fisher’s exact test for proportions or Kruskal–Wallis test for continuous variables. The denominator is as shown at the head of the column except where it was less in the case of e antigen status—one patient’s serum gave an indeterminate result.

### Large cell change

**Figure 2.**
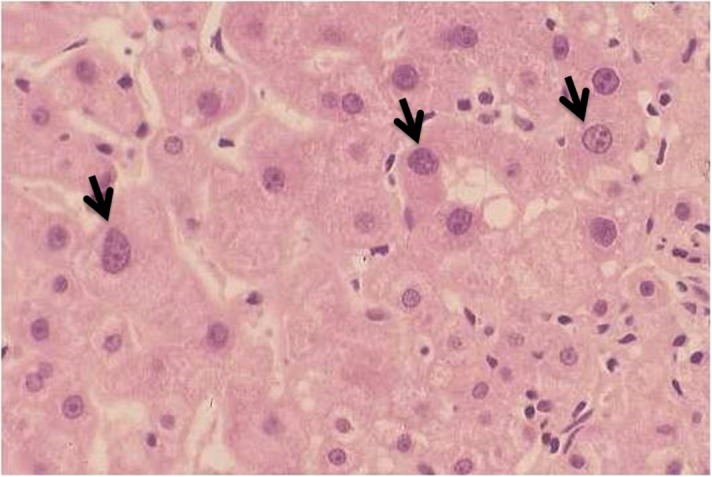
Histological appearances of large cell change in a liver biopsy from a Zambian man. In the upper part of the field there is a cluster of hepatocytes showing large cell change characterized by large nuclei in cells with increased cytoplasm. Haematoxylin and eosin–stained section (original magnification ×400).

**Figure 3.**
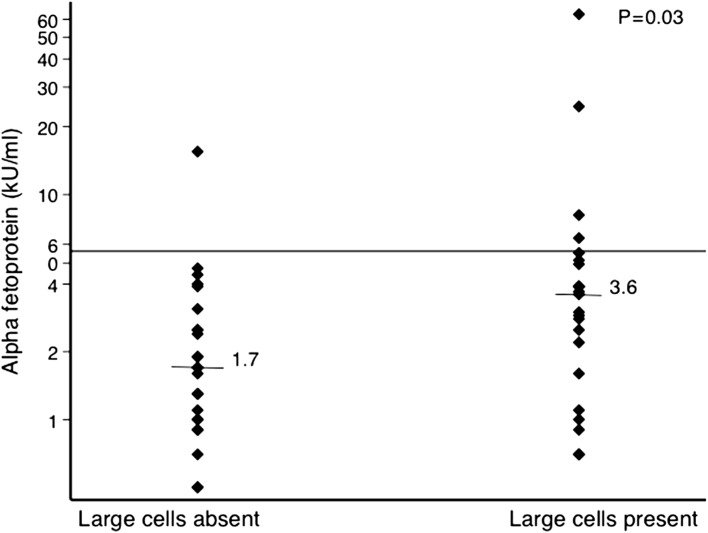
Serum α-protein concentrations (log scale) and large cell change.

Large cell change (Figure 2) was noted in 26 of 53 fully evaluated biopsies. Large cell change was clearly more common in biopsies showing higher stage and was also associated with Ishak score (Table 3). It was unrelated to age, sex, ALT, viral load, HBsAg concentration or HBe antigenaemia (Table 3). Large cell change was, however, associated with increased concentrations of α-fetoprotein, even though these were still within the normal range (Figure 3). No large cell change was identified in 30 necropsy samples from road traffic accidents in Zambia, and in a review of 100 biopsies from HBV-infected patients in London with various stages of disease, large cell change was observed in 5, 4 of which had cirrhosis.

## Discussion

Although there is wide variation in the precise estimates, there is no doubt that the global burden of disease due to HBV infection is huge, with estimates ranging from 240 million to 400 million infected people worldwide^11^ and with a considerable burden of cirrhosis and liver cancer.^2,8^ The prevalence of persistent HBV infection across Africa varies considerably, with estimates from Ghana of 12%^12^ and Zambian blood bank data suggesting a prevalence of 5.7%.^13^ However, there are few data on which to base prevention policies or treatment decisions in Africa. Here we show, in a cross-sectional analysis of a patient population selected to have some evidence of liver inflammation or damage, that there was already advanced disease in a significant proportion of patients, with two having evidence of cirrhosis and two cancers. Neither ALT nor GPR were fully able to identify patients with fibrosis and both tests would have missed patients who were in need of treatment. Large cell change was present in half of the patients biopsied and was associated with high-normal α-fetoprotein concentrations in blood.

The principal limitation of this study is its small size, as it was focused on liver biopsy, and it was therefore not powered to assess diagnostic accuracy. The data presented here are purely observational in nature. Self-report of alcohol intake is inaccurate, so we may have underestimated alcohol intake. However, none of the biopsies showed histological evidence of alcohol-mediated damage. The third limitation is the uncertainty about the most appropriate cut-off for ALT. We used a local rather than an international reference range. Nevertheless, it is apparent from Figure 1 that no value of ALT is perfectly discriminating and will always remain slightly arbitrary.

While the profile of disease in HIV-co-infected patients in Africa has been the subject of considerable interest,^14–16^ including children,^17^ paradoxically, fewer studies have been published about Africans with monoinfection. There are data to suggest that HBV acquisition in Zambia occurs horizontally in early childhood, rather than vertically,^18^ in common with other data from southern Africa.^19^ Vaccination programmes will reduce the overall burden of disease but will not immediately prevent vertically acquired transmission. HBV vaccination in Zambia is given as part of a pentavalent vaccine at 6, 10 and 14 weeks of age, although the World Health Organization recommends that the first dose be administered within 24 h after birth. The delayed first dose is a window of opportunity for transmission during the peri-partum period and in the early weeks of life.^20^ It seems likely that the early acquisition of infection during childhood potentiates the development of hepatocellular cancers in young men,^21^ but the reason for these particularly aggressive cancers has not yet been identified. It is possible that genetic factors play a part, but case ascertainment is poor, so an accurate family history is difficult to obtain.

The prevalence of HDV infection in this population appears to be <1%, consistent with previous estimates in this part of Africa.^22,23^ The prevalence was considerably higher (5%) in Nigeria.^24^

Quantitative HBsAg measurement reflects the transcription of closed circular DNA in the hepatocyte.^25^ We found no evidence that HBsAg concentrations were useful biomarkers of stage, inflammation or the need for treatment. Whether they predict later oncogenesis remains to be elucidated. This underlines the general point that biomarkers of disease activity and progression are urgently needed in Africa. Lemoine et al.^26^ found that the GPR was a useful biomarker of fibrosis, but we were unable to confirm this. Zambia and The Gambia are likely to have different patient populations for several reasons, including different ethnicity, the high prevalence of hepatosplenic schistosomiasis in Zambia and the widespread use of alcohol in Zambia, which would probably predict that biomarkers of liver disease would differ in the two countries.

The significance of large cell change is as yet unknown, but it was detected in half of our patients who had liver biopsies, in contrast to none of 30 road traffic accident victims (HBV status unknown) or 5% of patients with advanced HBV infection in London. Given that in southern Africa it is commonplace to diagnose hepatocellular cancer in men in their third decade,^2^ we must search for markers of malignant susceptibility in patients with persistent HBV infection, and this high prevalence is of concern. The change we have identified is characterized by increased cell size and nucleolar abnormalities, which necessarily raise the question as to the malignant potential of these changes and whether they can properly be classified as dysplastic or not. Further work is urgently needed to characterize the molecular changes that occur in these cells and then to follow up these patients carefully with regular monitoring. The significance of large cell change will ultimately determine how we stratify patients with HBV infection in Africa. If it is actually an innocent marker of regeneration, then our data suggest that ALT, viral load and possibly GPR may usefully contribute to identifying most of those patients who need treatment. If it is truly a biomarker of impending malignant change, then either novel biomarkers are needed or all patients should be treated with a view to cancer prevention. It is important to note that large cell change was observed more frequently in Zambia than in patients with chronic HBV infection in London. Many such patients in London have acquired their infection in Africa, so it remains a possibility that the large cell change observed in Zambia may be a consequence of synergy between HBV and some other environmental exposure.

## Supplementary data

Supplementary data are available at Transactions Online (http://trstmh.oxfordjournals.org/).

## Supplementary Material

Supplementary Data

## References

[1] Kew MC, Macerollo P Effect of age on the etiologic role of the hepatitis B virus in hepatocellular carcinoma in blacks. Gastroenterology. 1988;94(2):439–42. 2446950 10.1016/0016-5085(88)90434-9

[2] Carr BI, editor. Hepatocellular carcinoma: diagnosis and treatment. Totowa, NJ: Humana Press, 2016.

[3] Kew MC Hepatocellular carcinoma: epidemiology and risk factors. J Hepatocel Carcinoma. 2014;1:115–25. 10.2147/JHC.S44381 PMC4918271 27508181

[4] Marcellin P, Gane E, Buti M, et al Regression of cirrhosis during treatment with tenofovir disoproxil fumarate for chronic hepatitis B: a 5-year open-label follow-up study. Lancet. 2013;381(9865):468–75. 23234725 10.1016/S0140-6736(12)61425-1

[5] World Health Organization Guidelines for the prevention, care and treatment of persons with chronic hepatitis B infection. Geneva: World Health Organization, 2015. 26225396

[6] European Association for the Study of the Liver EASL clinical practice guidelines: management of chronic hepatitis B virus infection. J Hepatol. 2012;57(1):167–85. 22436845 10.1016/j.jhep.2012.02.010

[7] Terrault NA, Bzowej NH, Chang K-M, et al AASLD guidelines for treatment of chronic hepatitis B. Hepatology. 2016;63(1):261–83. 26566064 10.1002/hep.28156 PMC5987259

[8] Lemoine M, Eholié S, Lacombe K Reducing the neglected burden of viral hepatitis in Africa: strategies for a global approach. J Hepatol. 2015;62(2):469–76. 25457207 10.1016/j.jhep.2014.10.008

[9] Lemoine M, Shimakawa Y, Njie R, et al Acceptability and feasibility of a screen-and-treat programme for hepatitis B virus infection in The Gambia: the Prevention of Liver Fibrosis and Cancer in Africa (PROLIFICA) study. Lancet Glob Health. 2016;4(8):e559–67. 27443781 10.1016/S2214-109X(16)30130-9

[10] Mallory MA, Page SR, Hillyard DR Development and validation of a hepatitis B virus DNA sequencing assay for assessment of antiviral resistance, viral genotype and surface antigen mutation status. J Virol Methods. 2011;177(1):31–7. 21723325 10.1016/j.jviromet.2011.06.009

[11] Basnayake SK, Easterbrook PJ Wide variation in estimates of global prevalence and burden of chronic hepatitis B and C infection cited in published literature. J Viral Hepat. 2016;23(7):545–59. 27028545 10.1111/jvh.12519

[12] Ofori-Asenso R, Agyeman AA Hepatitis B in Ghana: a systematic review & meta-analysis of prevalence studies (1995–2015). BMC Infect Dis. 2016;16:130. 26987556 10.1186/s12879-016-1467-5 PMC4797341

[13] Oshitani H, Kasolo FC, Mpabalwani M, et al Prevalence of hepatitis B antigens in human immunodeficiency virus type 1 seropositive and seronegative pregnant women in Zambia. Trans R Soc Trop Med Hyg. 1996;90(3):235–6. 8758060 10.1016/s0035-9203(96)90227-8

[14] Wandeler G, Musukuma K, Zürcher S, et al Hepatitis B infection, viral load and resistance in HIV-infected patients in Mozambique and Zambia. PLoS One. 2016;11(3):e0152043. 27032097 10.1371/journal.pone.0152043 PMC4816321

[15] Archampong TN, Lartey M, Sagoe KW, et al Proportion and factors associated with Hepatitis B viremia in antiretroviral treatment naïve and experienced HIV co-infected Ghanaian patients. BMC Infect Dis. 2016;16:14. 26759172 10.1186/s12879-016-1342-4 PMC4710995

[16] Bivigou-Mboumba B, François-Souquière S, Deleplancque L, et al Broad range of hepatitis B virus (HBV) patterns, dual circulation of quasi-subgenotype A3 and HBV/E and heterogeneous HBV mutations in HIV-positive patients in Gabon. PLoS One. 2016;11(1):e0143869. 26764909 10.1371/journal.pone.0143869 PMC4713159

[17] Peebles K, Nchimba L, Chilengi R, et al Pediatric HIV-HBV coinfection in Lusaka, Zambia: prevalence and short-term treatment outcomes. J Trop Pediatr. 2015;61(6):464–7. 26338421 10.1093/tropej/fmv058 PMC4852213

[18] Tabor E, Bayley AC, Cairns J, et al Horizontal transmission of hepatitis B virus among children and adults in five rural villages in Zambia. J Med Virol. 1985;15(2):113–20. 3973567 10.1002/jmv.1890150203

[19] Botha JF, Ritchie MJ, Dusheiko GM, et al Hepatitis B virus carrier state in black children in Ovamboland: role of perinatal and horizontal infection. Lancet. 1984;1(8388):1210–2. 6144925 10.1016/s0140-6736(84)91694-5

[20] Keane E, Funk AL, Shimakawa Y Systematic review with meta-analysis: the risk of mother-to-child transmission of hepatitis B virus infection in sub-Saharan Africa. Aliment Pharmacol Ther. 2016;44(10):1005–17. 27630001 10.1111/apt.13795

[21] Shimakawa Y, Lemoine M, Njai HF, et al Natural history of chronic HBV infection in West Africa: a longitudinal population-based study from The Gambia. Gut. 2016;65(12):2007–16. 26185161 10.1136/gutjnl-2015-309892

[22] Cunha C, Tavanez JP, Gudima S Hepatitis delta virus: a fascinating and neglected pathogen. World J Virol. 2015;4(4):313–22. 26568914 10.5501/wjv.v4.i4.313 PMC4641224

[23] Winter A, Letang E, Vedastus Kalinjuma A, et al Absence of hepatitis delta infection in a large rural HIV cohort in Tanzania. Int J Infect Dis. 2016;46:8–10. 26996457 10.1016/j.ijid.2016.03.011

[24] Opaleye OO, Japhet OM, Adewumi OM, et al Molecular epidemiology of hepatitis D virus circulating in southwestern Nigeria. Virol J. 2016;13:61. 27044424 10.1186/s12985-016-0514-6 PMC4820959

[25] Boyd A, Maylin S, Moh R, et al Hepatitis B surface antigen quantification as a predictor of seroclearance during treatment in HIV-hepatitis B virus coinfected patients from sub-Saharan Africa. J Gastroenterol Hepatol. 2016;31(3):634–44. 26313291 10.1111/jgh.13156

[26] Lemoine M, Shimakawa Y, Nayagam S, et al The gamma-glutamyl transpeptidase to platelet ratio (GPR) predicts significant liver fibrosis and cirrhosis in patients with chronic HBV infection in West Africa. Gut. 2016;65:1369–76. 26109530 10.1136/gutjnl-2015-309260 PMC4975834

