# Supplementary material for: Stage of disease in hepatitis B virus infection in Zambian adults is associated with large cell change but not well defined using classic biomarkers
Source: Trans R Soc Trop Med Hyg. 2018 Jan 25;111(9):425–32. doi: 10.1093/trstmh/trx077 (PMC5914341; doi:10.1093/trstmh/trx077)
Supplement: Supplementary Data [file supmaterial.docx]

**Supplementary Table 1**

**Recruitment Flow Diagram**

Total enrolled

(n = 347)

HBsAg positive

Excluded (n = 243)

Did not meet inclusion criteria

Included (n = 104)

Met inclusion criteria

(ALT>35 and HBV DNA>2000IU/mL)

Total not biopsied (n = 36)

- High INR (n = 5)
- Low HBV DNA on retesting (n = 29)
- Declined (n = 2)

Total biopsied (n = 68)

(α-fetoprotein)

Biopsies available (n = 68)

**Reasons for not performing liver biopsy in 35 patients with abnormal ALT or transient elastography > 5kPa**

| Reason | n |
| --- | --- |
| Lost to follow up before biopsy scheduled | 5 |
| Low viral load, so biopsy felt unlikely to contribute to clinical decision-making | 10 |
| High INR | 6 |
| Treated at another institution with tenofovir/emtricitabine | 1 |
| High a-fetoprotein diagnostic of hepatocellular carcinoma | 1 |
| Died before biopsy scheduled | 1 |
| Declined | 2 |
| Menghini biopsy needle unavailable before treatment decision had to be made | 9 |
| Total | 35 |

**Supplementary Table 2**

**Baseline characteristics in relation to eligibility for therapy**

|  | Eligible for consideration of therapy (ALT>35 *and* DNA >2000 iu/l) | Ineligible for therapy | *P* |
| --- | --- | --- | --- |
| n | 40 | 65 |  |
| Sex (M:F) | 36:4 | 53:12 | 0.28 |
| Age (median, IQR) | 28 (23-34) | 31 (25-42) | 0.07 |
| eAg seropositive | 20/39 | 5/65 | <0.001 |
| HBsAg concentration | 10385 (5345-14310) | 6132 (2565-15690) | 0.21 |
| Fibroscan (kPa) | 7 (5.9,18.9) (n=12) | 6.2 (5.7-7.2) | 0.09 |
| Proportion with Fibroscan >5kPa | 11/12 | 47/52 | 1.00 |
| Proportion with Fibroscan >7.2kPa | 5/12 | 12/52 | 0.28 |
| γ-glutamyl transferase | 56 (37-112) | 34 (23-58) | 0.002 |
| GPR | 0.29 (0.19,0.54) | 0.18 (0.10,0.33) | 0.001 |
| High GPR (>0.32) | 30/36 (83%) | 29/55 (53%) | 0.003 |
| Ishak | 5.5 (3-7) | 3 (2-4) | 0.01 |
| Stage (need to categorise) | 3,4,5,5,3,1 | 8,10,11,1,1,0 | 0.06 |
| α−feto protein | 2.8 (2.0-4.0) | 1.9 (1.0-3.7) | 0.01 |

Eligibility was assessed as patients with ALT >1.3 times upper limit of normal (35 iu/l in this hospital) and viral load > 2000 iu/l.

**Supplementary Table 3**

**Baseline characteristics, by transient elastography**

| Fibroscan stiffness | Less than 5kPa (n=4) | 5kPa or more (n=60) | *P* | Less than 7.2kPa  (n=47) | 7.2kPa or more  (n=17) | *P* |
| --- | --- | --- | --- | --- | --- | --- |
| Sex (M:F) | 3:1 | 55:5 | 0.33 | 42:5 | 16:1 | 1.00 |
| Age (yrs; median, IQR) | 29 (26-32) | 31 (24-41) | 0.78 | 31 (25-42) | 26 (23-32) | 0.21 |
| BMI (kg/m^2^) (median, IQR) | 24.9 (22.6-25.3) | 22.2 (20.4-25.0) | 0.40 | 23.0 (20.3-25.7) | 21.6 (20.6-22.6) | 0.21 |
| Alcohol use currently | 0 | 11 | 1.00 | 9 | 2 | 0.71 |
| eAg positive | 0 | 12 | 1.00 | 7 | 5 | 0.28 |
| Schistosomiasis | 0 | 3 | - | 3 | 0 | - |
| Delta Ag positive | 0 | 1 |  | 1 | 0 |  |
| Platelet count (x10^9^/l) | 240 (227-311) | 210 (172-262) | 0.09 | 216 (176-263) | 205 (148-243) | 0.29 |
| ALT (i.u./l) | 51 (47-243) | 29 (21-40) | 0.02 | 29 (24-40) | 29 (20-54) | 0.98 |
| Viral load (log units/ml) | 2.5 (1.7-2.8) | 2.9 (2.4-4.2) | 0.15 | 2.9 (2.4-4.1) | 2.6 (2.4-4.2) | 0.85 |
| Genotype | 1 A so far | 2 E so far |  | 1 A | 2 E |  |

The manufacturer’s brochure (Echosens) recommends the cut-off of 7.2kPa for detection of fibrosis in HBV infection. The cut-off of 5kPa was also used as there is some suggestion that a more conservative threshold value my be useful for detection of minimal fibrosis.
